# Design and Evaluation of a Novel Multiplex Real-Time PCR Melting Curve Assay for the Simultaneous Detection of Nine Sexually Transmitted Disease Pathogens in Genitourinary Secretions

**DOI:** 10.3389/fcimb.2019.00382

**Published:** 2019-11-12

**Authors:** Xiao-Mei Hu, Jiang-Xia Xu, Li-Xia Jiang, Lian-Rui Deng, Zhen-Mei Gu, Xiao-Ying Xie, Hui-Cai Ji, Wei-Hua Wang, Li-Ming Li, Cheng-Nan Tian, Fang-Li Song, Shao Huang, Lei Zheng, Tian-Yu Zhong

**Affiliations:** ^1^Department of Laboratory Medicine, First Affiliated Hospital of Gannan Medical University, Ganzhou, China; ^2^Department of Precision Medicine Center, First Affiliated Hospital of Gannan Medical University, Ganzhou, China; ^3^Department of Medical Laboratory, The Fourth Affiliated Hospital of Nanchang University, Nanchang, China; ^4^Department of Laboratory Medicine Center, Nanfang Hospital, Southern Medical University, Guangzhou, China; ^5^Department of Obstetrics and Gynecology, First Affiliated Hospital of Gannan Medical University, Ganzhou, China; ^6^Department of Dermatology, First Affiliated Hospital of Gannan Medical University, Ganzhou, China; ^7^Department of Cardiac and Thoracic Surgery, First Affiliated Hospital of Gannan Medical University, Ganzhou, China; ^8^Jiangxi Shiningmed Medical Technology Ltd., Ganzhou, China

**Keywords:** multiplex, polymerase chain reaction, sanger sequencing, sexually transmitted diseases, pathogen

## Abstract

**Background:** Sexually transmitted diseases (STD) are a major cause of infertility, long-term disability, ectopic pregnancy, and premature birth. Therefore, the development of fast and low-cost laboratory STD diagnostic screening methods will contribute to reducing STD-induced reproductive tract damage and improve women's health worldwide. In this study, we evaluated a novel multiplex real-time PCR melting curve assay method for the simultaneous detection of 9 STD pathogens, including *Chlamydia trachomatis, Neisseria gonorrhoeae, Mycoplasma genitalium, Trichomonas vaginalis, Mycoplasma hominis, Ureaplasma urealyticum, Ureaplasma parvum*, and *herpes simplex* virus.

**Methods:** The analytical performance of the method, including its limit of detection (LOD), specificity, repeatability, and effect on different DNA extraction kits were evaluated. Additionally, we obtained 1,328 clinical specimens from 3 hospitals to detect the 9 STD pathogens using multiplex real-time PCR melting curve and Sanger sequencing, to evaluate the sensitivity, specificity, and consistency of the assay method.

**Results:** The results showed that the analytical sensitivity of the novel multiplex real-time PCR melting curve assay is very excellent, with LOD of DNA corresponding to <200 copies/μL for the DNA of the 9 STDs and 1.00 × 10^4^ color change unit /ml for those of UU and UP. Additionally, this assay demonstrated excellent analytical specificity, excellent repeatability, and its results had no effect of different DNA extraction kits. The performance, in terms of sensitivity (91.06–100%) and specificity (99.14–100%), was remarkable, since the consistency between it and Sanger sequencing was more than 0.85 in the clinic.

**Conclusion:** The novel multiplex real-time PCR melting curve assay method has high sensitivity and specificity, relatively low cost, and simple to use for the simultaneous detection of 9 STD pathogens in genitourinary secretions.

## Introduction

Sexually transmitted diseases (STD) are a major cause of infertility, long-term disability, ectopic pregnancy, and premature birth. They increase the risk of developing genital cancers, and are a severe medical, social, and economic burden to thousands of adults and babies worldwide (Warr et al., 2019). To date, more than 30 pathogens such as bacteria, viruses, and parasites have been shown to be transmitted through sexual contact. *Chlamydia trachomatis, Neisseria gonorrhoeae, Mycoplasma genitalium, Trichomonas vaginalis, Mycoplasma hominis, Ureaplasma urealyticum, Ureaplasma parvum, herpes simplex virus* (HSV) are reportedly the most prevalent STD pathogens (Kim et al., 2017). Some STD pathogens are cured using appropriate antibiotic treatment. However, most STD pathogens have atypical symptoms, are difficult to diagnose (Hilbert and Reno, 2018). Therefore, the development of fast and low-cost laboratory STD diagnostic screening methods will contribute to reducing STD-induced reproductive tract damage and improve women's health worldwide.

Sanger sequencing has highly accurate for diagnosing STD pathogens whereas has several limitations, including long sequencing/diagnosis time, high cost, and complex operation and result analysis. Additionally, in Sanger sequencing, a single reaction can only detect one pathogen. However, STD pathogens often co-infect their host (Ashshi et al., 2015; Kim et al., 2017). Therefore, methods enabling the simultaneous detection of multiple target pathogens are essential for accurate diagnosis. To date, multiplex polymerase chain reaction (PCR) has been found to be a highly sensitive method for detecting these sexually transmitted pathogens. The multiplex assay is also a cost-effective diagnostic test because it is labor-unintensive, has a low reagent cost, and enables faster detection (Lee et al., 2012; Rumyantseva et al., 2015).

This study therefore aimed at evaluating the sensitivity and specificity of a novel multiplex real-time quantitative PCR (real-time PCR) melting curve assay method, for the simultaneous detection of 9 STD pathogens, including *Chlamydia trachomatis, Neisseria gonorrhoeae, Trichomonas vaginalis*, HSVI, HSVII, *Mycoplasma genitalium, Ureaplasma urealyticum, Ureaplasma parvum*, and *Mycoplasma hominis*. multiplex real-time PCR based on probe-based fluorescence melting curve analysis (FMCA) which based on melting temperature generated by thermal denaturation of the probe-target hybrid (Huang et al., 2011). The basic principle as follows: Briefly, the probe labeled with a fluorescent group and a quenching group at both ends was added in the PCR system. The probe was bound to single-stranded oligonucleotide sequence complementary tois during the PCR process. After the completion of PCR process, the melting curve analysis process is added to carry out Thermal denaturation of hybrids formed between probes and PCR-synthetic oligonucleotide targets. Then fluorescence value was recorded in the corresponding detection channel. By calculating the negative derivative of the fluorescence value and the temperature, the melting curve of the hybrid product of the probe and the sequence can be obtained, and the melting point (Tm value) is obtained. The number of genes detectable can be further increased if multiple probes each labeled with a different fluorophore are used. We also used the Kappa test to evaluate the consistency of its results with those of Sanger sequencing.

## Materials and Methods

### Clinical Specimens

Clinical specimens (1,328), including genitourinary secretion cotton swabs and scrapings of urethral orifices, or diseased areas, were collected and tested at three clinical sites in Jiangxi province, China: (1) 620 clinical specimens were collected at Hospital A; (2) 328 clinical specimens were collected at Hospital B; and (3) 380 clinical specimens were collected at Hospital C. All clinical specimens were uniformly numbered. Clinical specimens were stored at −18°C for no more than 6 months.

### Performance Analysis

#### Real-Time PCR Melting Curve Assay

Real-time PCR was performed using the real-time PCR melting curve assay and the Rotor-Gene Q real-time PCR system (Qiagen, Hilden, Germany). Twenty microliter PCR reaction system: 2 × PCR 12.5 μL, 2.0 μL STD universal primer, 2.5 μL STDs specific primers, 2.0 μL STD Probes, 1.0 μL STD internal control primer, and then 5 μL of the target DNA was added, mixed, and amplified. Thermocycling conditions for nested qPCR were as follows: Initial denaturation at 95°C for 5 min, followed by 10 cycles at 95°C for 15 s, 55°C for 30 s, 72°C for 30 s, followed by 20 cycles at 95°C for 20 s, 72°C for 120 s, followed by 30 cycles at 95°C for 15 s, 60°C for 30 s, 72°C for 30 s. Then the Tm value were measured by dissociation curve. Purified water was used as a negative control and the 9 STD pathogens were used as positive controls. The primers and probes were designed against conserved genes within each pathogen (Tables 1, 2), Tm value was calculated using Tm Utility 1.5 (Idaho Technology, Inc., Salt Lake City, UT, USA). Each tube has an Internal Control to provide a valid “negative” result which have a melting peak at 76.0 ± 2°C in the HEX fluorescent channel. The representative images of melting curve analysis results were shown in Supplement Figure 1.

**Table 1:** Nucleotide sequences of preamplification primers.

**Target gene**	**Primers**	**Sequences**	**Tm (°C)**
Chlamydia trachomatis	SC-STD1-F	gggttccctaagggttggaGGGAATCCTGCTGAACCAA	61.0
	SC-STD1-R	gtgccagcaagatccaatctagaTCAAAACACGGTCGAAAACA	60.0
Neisseria gonorrheae	SC-STD3-F	gggttccctaagggttggaCGGCAGCATTCAATTTGTT	59.0
	SC-STD3-R	gtgccagcaagatccaatctagaAAAAAGCCGCCATTTTTGTA	58.5
Trichomonas vaginalis	SC-STD5-F	gggttccctaagggttggaCCAGAAGTGGGCTACACACC	63.0
	SC-STD5-R	gtgccagcaagatccaatctagaATACCAAGGCCGGAAGCAC	64.0
Herpes simplex virus II	SC-STD7-F	gggttccctaagggttggaCATGGGGCGTTTGACCTC	62.4
	SC-STD7-R	gtgccagcaagatccaatctagaTACACAGTGATCGGGATGCT	62.2
Mycoplasma genitalium	SC-STD4-F	gggttccctaagggttggaACCTTGATGGTCAGCAAAACTT	62.0
	SC-STD4-R	gtgccagcaagatccaatctagaCCTTTGATCTCATTCCAATCAGTA	59.5
Herpes simplex virus I	SC-STD6-F	gggttccctaagggttggaCTGTGGTGTTTTTGGCATCA	60.7
	SC-STD6-R	gtgccagcaagatccaatctagaGGTTGTGGAGGAGACGTTG	61.8
Ureaplasma urealyticum /Ureaplasma parvum	SC-STD9/10-F	gggttccctaagggttggaGCTGACGTTGCAAGAAGACG	63.6
	SC-STD9/10-R	gtgccagcaagatccaatctagaACCATCAGGGAAAGTAACTTCAAC	62.0
Internal control	SC-IAC-F	gggttccctaagggttggaACATGTAACCGCCCCCATT	63.2
	SC-IAC-R	gtgccagcaagatccaatctagaTCCACGCACGCACTACTATG	63.7
Mycoplasma hominis	SC-MH–F	gggttccctaagggttggaGGAAGATATGTAACAAAAGAAGGTGCTG	63.3
	SC-MH-R	gtgccagcaagatccaatctagaTTTATCTTCTGGCGTAATGATATCTTCG	62.2
General primer	Primer-F	gggttccctaagggttgga	62.2
	Primer-R	gtgccagcaagatccaatctaga	63.6

*Lowercase letters indicate general primer, uppercase letters indicate specific primer. Tm value is calculated using only the specific primer portion, excluding the general primer portion*.

**Table 2:** Nucleotide sequences of pathogen-specific regions of the probes.

**Probes**	**Target gene**	**Sequences**	**Channels**	**Tm (°C)**
SC-MH-HEX	Mycoplasma hominis	ACTGCTCCAGCTAAAAGCGAA	557 ± 5	62.5
SC-STD5-HEX	Trichomonas vaginalis	TCTGGCAAGATCAAGGACATCCTCC	557 ± 5	67.0
SC-STD9/10-ROX	Ureaplasma urealyticum/ Ureaplasma parvum	CTTTAATTACTGATCATGTAATGGAAGGGGCA	610 ± 5	56.5/66.0
SC-STD7-ROX	herpes simplex virus II	TGAAGCGTGTTTACCACATTCAGCCGAGCCTG	610 ± 5	74.0
SC-STD3-FAM	Neisseria gonorrheae	AGTAATCAGATGAAACCAG	510 ± 5	54.5
SC-STD4-FAM	Mycoplasma genitalium	AGCCTATCTTTGATCCTTTTAAAGGCTTTGG	510 ± 5	66.0
SC-STD6-Cy5	herpes simplex virus I	TTATCCCATTCCTTTTGGTTCTTGTCGGTG	660 ± 10	68.5
SC-STD1-Cy5	Chlamydia trachomatis	CGCTATCAGCATGCGT	660 ± 10	57.5
SC-IAC-HEX	Internal control	GAGAGGCCGCACCTTGGTAGTAAATAGACACATGGCCGAGT	557 ± 5	76.0

*Tm value is calculated using the full length of the probe*.

#### Establishing the Limit of Detection (LOD)

To establish the LOD for each pathogen, nine STD pathogens of enterprise reference panel (plasmid, National institutes for food and drug control of China, Beijing, China) and nine STD pathogens of enterprise reference panel (positive clinical specimens, National institutes for food and drug control of China) were used. The plasmids of the STD pathogens were diluted with TE buffer to 10^6^, 10^5^, 10^4^, 10^3^, 10^2^ copies/μL, then measured using the real-time PCR melting curve assay and analyzed Ct value. Then standard curve was drawn based on Ct value and plasmids concentration. Additionally, DNA from clinical specimens were measured using the real-time PCR melting curve assay and analyzed Ct value, then the sample concentration was calculated according to standard curve. Finally, DNA from clinical specimens were diluted with TE buffer to 400 copies/μL, 200 copies/μL, and 100 copies/μL, then measured using the real-time PCR melting curve assay. There were 20 portions of each concentration. All assays were performed in triplicates. The lowest concentration at which at least 19 of the 20 test results were positive was the LOD.

#### Determination of Specificity

To determine whether our novel assay method can produce non-specific reactions with other homologous genes or negative specimens, which will affect the judgment of clinical test results, it was used to analyze five healthy human secretion specimens and other pathogen specimens. The pathogen specimens included specimens of *treponema pallidum, candida albicans, E. coli*, HPV16, 18, 31, 33, 35, 39, 45, 51, 52, 56, 58, 59, 66, and 68 (National institutes for food and drug control of China). All assays were performed in triplicates. Then, method specificity was evaluated based on negative or positive test results.

#### Determination of Sensitivity

To evaluate its sensitivity to each pathogen, nine STD pathogens of enterprise reference panel (plasmid, National institutes for food and drug control of China) and nine STD pathogens of enterprise reference panel (clinical specimens, National institutes for food and drug control of China) were used. The plasmids and clinical specimens were measured using the real-time PCR melting curve assay. All assays were performed in triplicates. Then, method sensitivity was evaluated based on negative or positive test results.

#### Determination of Repeatability

To evaluate method repeatability, nine STD pathogens of enterprise reference panel (plasmid, National institutes for food and drug control of China) and nine STD pathogens of enterprise reference panel (positive clinical specimens, National institutes for food, and drug control of China) were detected using the real-time PCR melting curve assay. Briefly, the plasmids of STD pathogens were quantified and diluted with TE buffer to 2,000 copies/μL and 200 copies/μL, then measured using the real-time PCR melting curve assay. There were 10 portions of each concentration. We evaluated the repeatability of the method based on the coefficient of variation (CV) of the Tm value.

#### Effects of Different DNA Extraction Kits on Test Results

DNA in positive and negative STD specimens were extracted using TIANamp Bacteria DNA Kit (TIANGEN, Beijing, China), AxyPrep Bacterial Genomic DNA Miniprep Kit (Axygen, Union City, CA, Corning, USA), and Bacteria Genomic DNA Kit (CWbio, Beijing, China), according to the manufacturer's protocol. Then, the STD pathogens were analyzed using the real-time PCR melting curve assay. The effects of different DNA extraction kits on test results were evaluated based on the test results.

### Clinical Performance

#### Sample Processing

The specimens were rinsed by drawing 1.0 mL of sterile physiological saline, centrifuging at 12,000 × g for 10 min, and then collecting the precipitate. DNA was then extracted using the TIANamp Bacteria DNA Kit (TianGen) and stored at −20°C for at most 6 months. The samples were then processed and tested by independent operators who were unaware of sample contents, using Sanger sequencing and the real-time PCR melting curve assay.

#### Sanger Sequencing

Firstly, 20 μL of the sequencing solution was configured: 12.55 μL RNase Free Water, 5.0 μL 5 × GoldStar PCR Buffer, 0.2 μL 25 mM dNTP Mixture, 0.25 μL GoldStar DNA Polymerase, 1.0 μL 10 μM forward primer, and 1.0 μL 10 μM reverse primer, and then 5 μL of the target DNA was added, mixed, and amplified. The cycle parameters were initial denaturation at 95°C for 5 min; followed by 30 cycles at 95°C for 15 s, 55°C for 30 s, 72°C for 1 min, and then at 72°C for 10 min. A negative and positive control was set. The PCR product was sequenced using BGI (Beijing, China). The primers were designed against conserved genes within 9 STD pathogens (Supplementary Table 1).

### Statistical Analyses

Using the Sanger sequencing results as the control group, the Stata 12.0 software was used to calculate the sensitivity and specificity of the assay method, and the 95% confidence interval. The consistency of the method's test results with those of Sanger sequencing was evaluated using the Kappa test method with the SPSS software.

## Results

### Analytical Performance

#### LOD

STD pathogens enterprise reference panel (plasmids and clinical specimens at concentrations of 400 copies/μL, 200 copies/μL, and 100 copies/μL were verified using the real-time PCR melting curve assay, each 20 times. There were 19/20 positive results at the concentration of 100 copies/μL. Whereas, at the concentration of 400 copies/μL and 200 copies/μL, positive results were no <19/20 s. Therefore, the analytical LOD of the DNAs of the 9 STD pathogens in this method is 200 copies/μL. Additionally, the analytical LOD of the DNAs of *Ureaplasma urealyticum* and UP were 1.00× 10^4^ color change unit (CCU)/ml.

#### Analytical Specificity

Five healthy human secretion specimens and other pathogen specimens, including specimens of *treponema pallidum, candida albicans, E. coli*, HPV16, 18, 31, 33, 35, 39, 45, 51, 52, 56, 58, 59, 66, 68 were tested thrice using the novel assay method. The results were negative (data not shown) and cross-reactivity was not observed. These results show that the method specificity is very high.

#### Analytical Sensitivity

Nine STD pathogens of enterprise reference panel (plasmids and clinical specimens) were verified using the real-time PCR melting curve assay. The results showed that the positive rate was 100%. Tests for all pathogens were positive.

#### Analytical Repeatability

Nine STD pathogens of enterprise reference panel (plasmids and clinical specimens) at concentrations of 2,000 and 200 copies/μL were verified using the real-time PCR melting curve assay, each 10 times. Tests for all pathogens were positive. Additionally, the in-batch CV was no more than 5%, with excellent repeatability (Supplementary Table 2).

#### Effects of Different DNA Extraction Kits on Test Results

DNA extracted using DNA isolation kits from TIANGEN (Beijing, China), Axygen biosciences (Corning, USA), and CWbio (Beijing, China) were analyzed using the real-time PCR melting curve assay. The results showed that the results obtained using all the DNA extraction kits were excellently consistent (Supplementary Table 3).

### Clinical Performance

We used 5 μL 100 copies/μL of the STD pathogens plasmid as a template for amplification and sequencing, and all of them obtained positive results. The prevalence of *Chlamydia trachomatis, Neisseria gonorrhoeae, Trichomonas vaginalis*, HSVI, HSVII, *Mycoplasma genitalium, Ureaplasma urealyticum, Ureaplasma parvum*, and *Mycoplasma hominis* in the 1,328 samples were 8.06% (107 cases), 2.79% (37 cases), 2.71% (36 cases), 2.94% (39 cases), 6.40% (85 cases), 2.03% (27 cases), 9.94% (132 cases), 37.80% (502 cases), and 9.04% (120 cases), respectively. The clinical performance of the assay method in three hospitals is summarized in Tables 3–5, while its total clinical performance is summarized in Table 6. Concordance between results of the assay method and those of Sanger sequencing was more than 0.85 (Kappa test, *P* < 0.001). The assay method was also highly sensitive and specific. Additionally, using this multiplex real-time PCR melting curve assay, nine STD pathogens could be detected within 3 h with limited hands-on time (Not include the DNA extraction). Finally, we found that the cost of PCR + Sanger sequencing to detect and identify 9 STD pathogens is about $20 per person, while multiplex real-time PCR melting curve assay requires only $3 in China.

**Table 3:** Performance of novel multiplex real-time PCR assay compared to Sanger sequencing in Hospital A.

**Pathogene**	**Sensitivity (95% CI)**	**Specificity (95% CI)**	**Kappa value**	**Kappa *P***	**McNemer *P***
*Chlamydia trachomatis*	99.49% (99.49–100.15%)	92.73% (90.68–94.77%)	0.949	<0.001	0.375
*Neisseria gonorrhoeae*	100% (100–100%)	96.00% (94.46–97.54%)	0.979	<0.001	1.000
*Trichomonas vaginalis*	100% (100–100%)	96.00% (94.46–97.54%)	0.979	<0.001	1.000
HSVI	100% (100–100%)	99.84% (99.52–100.15%)	0.855	<0.001	1.000
HSVII	98.15% (97.09–99.21%)	99.47% (98.09–100.04%)	0.960	<0.001	0.625
*Mycoplasma genitalium*	94.44% (92.64–96.25%)	100% (100–100%)	0.971	<0.001	1.000
*Ureaplasma urealyticum*	88.89% (86.42–91.36%)	99.41% (98.81–100.01%	0.913	<0.001	0.035
*Ureaplasma parvum*	93.81% (91.91–95.71%)	99.51% (98.96–100.06%	0.945	<0.001	0.007
*Mycoplasma hominis*	90.41% (88.09–92.73%)	100% (100–100%)	0.943	<0.001	0.016

**Table 4:** Performance of novel multiplex real-time PCR assay compared to Sanger sequencing in Hospital B.

**Pathogene**	**Sensitivity (95% CI)**	**Specificity (95% CI)**	**Kappa value**	**Kappa *P***	**McNemer *P***
*Chlamydia trachomatis*	100% (100–100%)	100% (100–100%)	1.000	<0.001	1.000
*Neisseria gonorrhoeae*	100% (100–100%)	100% (100–100%)	1.000	<0.001	1.000
*Trichomonas vaginalis*	100% (100–100%)	100% (100–100%)	1.000	<0.001	1.000
HSVI	100% (100–100%)	100% (100–100%)	1.000	<0.001	1.000
HSVII	100% (100–100%)	100% (100–100%)	1.000	<0.001	1.000
*Mycoplasma genitalium*	100% (100–100%)	100% (100–100%)	1.000	<0.001	1.000
*Ureaplasma urealyticum*	100% (100–100%)	100% (100–100%)	1.000	<0.001	1.000
*Ureaplasma parvum*	99.32% (98.42–100.21%)	98.35% (96.97–99.73%)	0.975	<0.001	0.625
*Mycoplasma hominis*	100% (100–100%)	100% (100–100%)	1.000	<0.001	1.000

*Trichomonas vaginalis*.

**Table 5:** Performance of novel multiplex real-time PCR assay compared to Sanger sequencing in Hospital C.

**Pathogene**	**Sensitivity (95% CI)**	**Specificity (95% CI)**	**Kappa value**	**Kappa *P***	**McNemer *P***
*Chlamydia trachomatis*	100% (100–100%)	100% (100–100%)	1.000	<0.001	1.000
*Neisseria gonorrhoeae*	100% (100–100%)	100% (100–100%)	1.000	<0.001	1.000
*Trichomonas vaginalis*	100% (100–100%)	100% (100–100%)	1.000	<0.001	1.000
HSVI	100% (100–100%)	99.73% (99.20–100.25%)	0.959	<0.001	1.000
HSVII	100% (100–100%)	100% (100–100%)	1.000	<0.001	1.000
*Mycoplasma genitalium*	100% (100–100%)	100% (100–100%)	1.000	<0.001	1.000
*Ureaplasma urealyticum*	97.83% (96.36–99.29%)	99.40% (98.63–100.18%)	0.963	<0.001	1.000
*Ureaplasma parvum*	97.48% (95.91–99.06%)	99.10% (98.14–100.05%)	0.968	<0.001	0.687
*Mycoplasma hominis*	87.88% (84.60–91.16%)	100% (100–100%)	0.930	<0.001	0.125

*Trichomonas vaginalis*.

**Table 6:** Performance of novel multiplex real-time PCR assay compared to Sanger sequencing in all Clinical specimens.

**Pathogene**	**Sensitivity (95% CI)**	**Specificity (95% CI)**	**Kappa value**	**Kappa *P***	**McNemer *P***
*Chlamydia trachomatis*	99.72% (99.25–100.20%)	96.26% (95.54–97.98%)	0.974	<0.001	0.375
*Neisseria gonorrhoeae*	100% (100–100%)	96.37% (96.51–98.23%)	0.980	<0.001	1.000
*Trichomonas vaginalis*	100% (100–100%)	99.92% (99.77–100.07%)	0.986	<0.001	1.000
HSVI	97.94% (96.59–98.29%)	99.77% (99.51–100.03%)	0.948	<0.001	0.625
HSVII	98.82% (98.24–99.40%)	99.76% (99.49–100.02%)	0.975	<0.001	0.625
*Mycoplasma genitalium*	96.30% (95.28–97.31%)	100% (100–100%)	0.981	<0.001	1.000
*Ureaplasma urealyticum*	92.86% (91.47–94.24%)	99.56% (99.21–99.92%	0.942	<0.001	0.096
*Ureaplasma parvum*	96.50% (95.52–97.49%)	99.14% (98.64–99.64%	0.959	<0.001	0.043
*Mycoplasma hominis*	91.06% (89.52–92.59%)	100% (100–100%)	0.949	<0.001	0.001

## Discussion

Clinical specimens from STD patients frequently contain multiple pathogens, and multiplex PCR has considerable potential for the much needed rapid, accurate, and simultaneous diagnosis of these pathogens. Multiplex PCR permits the use of more than a single set of primers, leading to simultaneous amplification of multiple sequences under a single reaction (Markoulatos et al., 2002). This study evaluated a novel multiplex PCR assay for the simultaneous detection of 9 potential STD pathogens, including *Chlamydia trachomatis, Neisseria gonorrhoeae, Trichomonas vaginalis*, HSVI, HSVII, *Mycoplasma genitalium, Ureaplasma urealyticum, Ureaplasma parvum*, and *Mycoplasma hominis*. We found that the consistency between the results of the novel assay method and those of Sanger sequencing was more that 0.85 (Kappa test, *P* < 0.001), and that the assay was highly sensitive and specific.

The analytical performance of every new testing method must be rigorously evaluated. Previous studies have reported several drawbacks of multiplex PCR assays. For example, PCR drift, competitive inhibition, and non-specific interactions reportedly caused multiplex assays to exhibit lower sensitivities than conventional PCR assays (Kweon et al., 2015). In this study, we found that the analytical sensitivity of the novel multiplex assay is very excellent, with the LOD of DNA corresponding to <200 copies/μL for 9 STD pathogens, and 1.00 × 10^4^ CCU/ml for *Ureaplasma urealyticum* and *Ureaplasma parvum*. Additionally, this assay demonstrated excellent analytical specificity and no cross-reactivity with specimens of secretions from five healthy human and other pathogen, including *treponema pallidum, candida albicans, E. coli*, HPV16, 18, 31, 33, 35, 39, 45, 51, 52, 56, 58, 59, 66, and 68. Finally, the assay demonstrated excellent repeatability and the results do not depend on the extraction kit used DNA extraction kits. These results therefore suggest that this novel assay method has sufficiently sensitivity, specificity, and repeatability in the detection of the 9 STD pathogens.

Broad-spectrum antibiotics are generally recommended for the treatment of STDs because of their diverse pathogen content (Workowski and Berman, 2010). However, some pathogens are not sensitive or are resistant to broad-spectrum antibiotics (Workowski and Berman, 2010; Schwebke et al., 2011). Successful identification of pathogens contributes to the selection of an optimal treatment option (Kim et al., 2014). Kim et al. (2017) confirmed the ability of the multiplex PCR method to detect *Neisseria gonorrhoeae, Chlamydia trachomatis, Ureaplasma urealyticum, Mycoplasma genitalium, Mycoplasma hominis*, and HSV II and *Trichomonas vaginalis* with a 96% sensitivity and 98% specificity. The **A**nyplex™ II STI-7 Detection kit (Seegene, Korea) exhibited high sensitivity in the detection of *Chlamydia trachomatis, Neisseria gonorrhoeae, Mycoplasma genitalium, Mycoplasma hominis, Ureaplasma urealyticum, Ureaplasma parvum*, and *Trichomonas vaginalis* in swabs (Bercot et al., 2015; Fernandez et al., 2016). The Real-Q STIs Kit assay can simultaneously detect six STD pathogens. It showed 100% sensitivity and specificity in the detection of *Mycoplasma hominis, Mycoplasma genitalium, Chlamydia trachomatis, Trichomonas vaginalis*, and *Neisseria gonorrhoeae*, and 94.1% sensitivity and 100% specificity in the detection of *Ureaplasma urealyticum* (Kim et al., 2015). The FTD STD9 kit had high sensitivity and specificity for in detecting seven pathogens, including *Chlamydia trachomatis, Neisseria gonorrhoeae, Mycoplasma genitalium, Mycoplasma hominis, Ureaplasma urealyticum/Ureaplasma parvum, Trichomonas vaginalis*, and HSV-I/II (Ashshi et al., 2015). However, these kits are unable to either distinguish between *Ureaplasma urealyticum and Ureaplasma parvum* or the HSV genotypes. We further evaluated the sensitivity and specificity of the novel multiplex PCR assay using clinical specimens, and compared with that of Sanger sequencing. The Its performance, in terms of sensitivity (91.06–100%) and specificity (99.14–100%), of the multiplex PCR was very good, since the consistency between its results and those of Sanger sequencing was more that 0.85 (Kappa test, *P* < 0.001). Additionally, the method could distinguish between the*Ureaplasma urealyticum, Ureaplasma parvum*, and the HSV genotypes. Moreover, our assay methods showed 90.41–100%, 99.32–100%, and 88.78–100% sensitivity for in detecting nine STD pathogens from three hospitals. Additionally, it showed 92.73–100%, 98.35–100%, and 99.10–100% specificity for in detecting nine STD pathogens from three hospitals. These results indicate that personnel operation ability has little effect on the results of this novel assay method. Additionally, the decreased sensitivity of our assay for *Mycoplasma hominis* and *Ureaplasma urealyticum* compared to other analytes. Possible reason was as follow: Sanger sequencing is that each STD pathogen is sequenced separately, whereas the multiplex real-time PCR melting curve assay is a reaction to detect nine pathogens. The sensitivity of the Sanger sequencing is higher than multiplex real-time PCR melting curve assay, so the number of positive cases detected by the Sanger sequencing is higher than that of the multiplex real-time PCR melting curve assay. Again, the sensitivity comes from the preparatory PCR and not from the sequencing itself.

There are three limitations to the data in this study. First, we used the pathogen specimens included specimens of *Treponema pallidum, Candida albicans, E. coli*, and HPV to estimate the specificity of multiplex real-time PCR melting curve assay. Indeed, it will be more profound if we used microbes such as non-pathogenic Neisseria, non-pathogenic *Mycoplasma, Pentatrichomonas hominis, Chlamydia pneumonia*, and other “like” organisms rather than *E. coli* to assess specificity. Additionally, the decreased sensitivity of our assay for *Mycoplasma hominis* and *Ureaplasma urealyticum* compared to other analytes. The specific reasons need further investigation. Finally, assay results were different in three hospital but we have not investigated the issue at present. We will continue to investigate the clinical effects of the multiplex real-time PCR melting curve assay in order to develop novel test kits for clinical testing for STD pathogens.

In conclusion, our method has sufficiently analytical performance, including high sensitivity, specificity, and repeatability in the detection of the nine STD pathogens. It was also highly sensitive and specific in the clinical detection of the nine STD pathogens. Additionally, it is easy of use and can easily be incorporated into daily laboratory testing.

## Data Availability Statement

The datasets analyzed in this manuscript are not publicly available. Requests to access the datasets should be directed to T-YZ, zhongtianyu@126.com.

## Ethics Statement

This study was conducted according to 1983 revision of the principles of the Helsinki Declaration of 1975, and was approved by the Ethics Committee of Gannan Medical University, The Fourth Affiliated Hospital of Nanchang University, and Nanfang Hospital, Southern Medical University. Three Ethics Committees granted this study an exemption from the informed consent requirement due to the use of residual de-identified patient specimens.

## Author Contributions

X-MH, J-XX, L-XJ, T-YZ, LZ, and SH designed the study. X-MH, J-XX, L-XJ, L-RD, Z-MG, X-YX, H-CJ, W-HW, L-ML, C-NT, and F-LS collected the data. L-RD, Z-MG, X-YX, H-CJ, W-HW, L-ML, C-NT, F-LS, and T-YZ analyzed the data. X-MH, J-XX, and L-XJ wrote the manuscript. T-YZ, LZ, and SH revised the manuscript. All authors read and approved the final manuscript.

### Conflict of Interest

F-LS and SH were employed by company Jiangxi Shiningmed Medical Technology Ltd. The remaining authors declare that the research was conducted in the absence of any commercial or financial relationships that could be construed as a potential conflict of interest.

## Supplementary Material

Supplement Figure 1 The representative images of melting curve analysis results. After the probe thermal dissociation from its target sequence, the fluorescent value (F) was recorded. Melting curve plots of fluorescence vs. temperature (T) are transformed into melting peaks by plotting –dF/dT vs. temperature. Each fluorescent detection probe has a fixed fluorescent channel and a Tm value. When the Tm value appears melting peaks, the result is positive, otherwise, the result is negative, or other non-specific products. **(A)** *Chlamydia trachomatis*; **(B)** *Neisseria gonorrhoeae*; **(C)** *Trichomonas vaginalis*; **(D)** HSVII; **(E)** *Mycoplasma genitalium*; **(F)** HSVI; **(G)** *Ureaplasma urealyticum /Ureaplasma parvum*; **(H)** *Mycoplasma hominis*.

Supplementary Table 1 The primers of 9 STD pathogens for Sangeb Sequencing.

Supplementary Table 2 Repeatability test result.

Supplementary Table 3 Effects of different DNA extraction kits on test results.

## Abbreviations

STD: Sexually transmitted diseases
HSV: herpes simplex virus
LOD: limit of detection.
