# Supplementary figures and images for: Design and Evaluation of a Novel Multiplex Real-Time PCR Melting Curve Assay for the Simultaneous Detection of Nine Sexually Transmitted Disease Pathogens in Genitourinary Secretions
Source: Front Cell Infect Microbiol. 2019 Nov 12;9:382. doi: 10.3389/fcimb.2019.00382 (PMC6861374; doi:10.3389/fcimb.2019.00382)

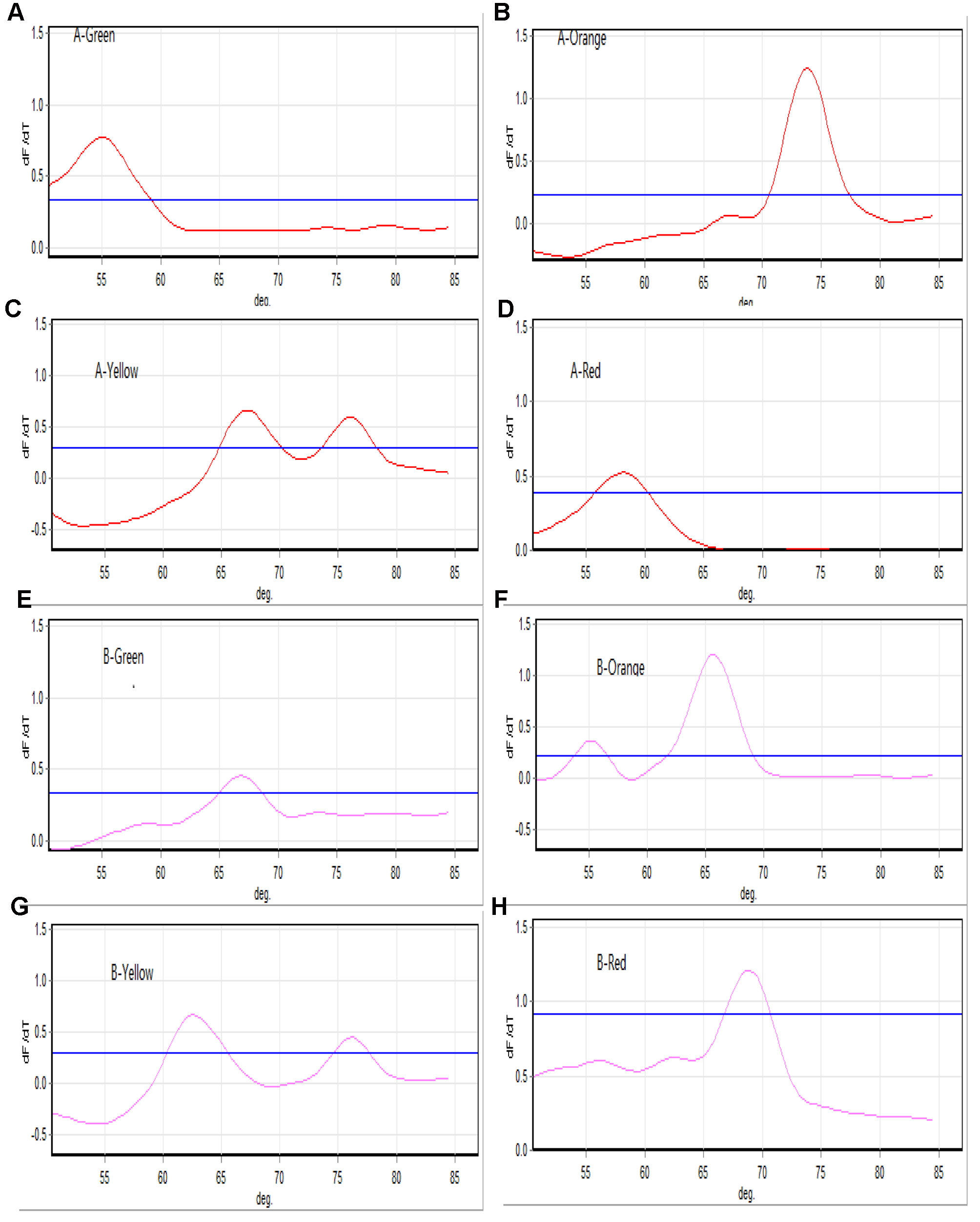

Supplement: Supplement Figure 1 — The representative images of melting curve analysis results. After the probe thermal dissociation from its target sequence, the fluorescent value (F) was recorded. Melting curve plots of fluorescence vs. temperature (T) are transformed into melting peaks by plotting –dF/dT vs. temperature. Each fluorescent detection probe has a fixed fluorescent channel and a Tm value. When the Tm value appears melting peaks, the result is positive, otherwise, the result is negative, or other non-specific products. (A) Chlamydia trachomatis; (B) Neisseria gonorrhoeae; (C) Trichomonas vaginalis; (D) HSVII; (E) Mycoplasma genitalium; (F) HSVI; (G) Ureaplasma urealyticum /Ureaplasma parvum; (H) Mycoplasma hominis. [file Image_1.TIF]
