# Supplementary material for: Design and Evaluation of a Novel Multiplex Real-Time PCR Melting Curve Assay for the Simultaneous Detection of Nine Sexually Transmitted Disease Pathogens in Genitourinary Secretions
Source: Front Cell Infect Microbiol. 2019 Nov 12;9:382. doi: 10.3389/fcimb.2019.00382 (PMC6861374; doi:10.3389/fcimb.2019.00382)
Supplement: Supplementary Table 1 — The primers of 9 STD pathogens for Sangeb Sequencing. [file Table_1.DOCX]

**Supplementary Table 1** The primers of 9 STD pathogens for Sanger Sequencing

| Primers | Target gene | Sequences |
| --- | --- | --- |
| STD1-F | Chlamydia trachomatis | GGGAATCCTGCTGAACCAA |
| STD1-R | Chlamydia trachomatis | TCAAAACACGGTCGAAAACA |
| STD3-F | Neisseria gonorrheae | CGGCAGCATTCAATTTGTT |
| STD3-R | Neisseria gonorrheae | AAAAAGCCGCCATTTTTGTA |
| STD5-F | Trichomonas vaginalis | CCAGAAGTGGGCTACACACC |
| STD5-R | Trichomonas vaginalis | ATACCAAGGCCGGAAGCAC |
| STD7-F | herpes simplex virus II | CATGGGGCGTTTGACCTC |
| STD7-R | herpes simplex virus II | TACACAGTGATCGGGATGCT |
| STD4-F | Mycoplasma genitalium | ACCTTGATGGTCAGCAAAACTT |
| STD4-R | Mycoplasma genitalium | CCTTTGATCTCATTCCAATCAGTA |
| STD6-F | herpes simplex virus I | CTGTGGTGTTTTTGGCATCA |
| STD6-R | herpes simplex virus I | GGTTGTGGAGGAGACGTTG |
| STD9/10-F | Ureaplasma urealyticum/ Ureaplasma parvum | GCTGACGTTGCAAGAAGACG |
| STD9/10-R | Ureaplasma urealyticum/ Ureaplasma parvum | ACCATCAGGGAAAGTAACTTCAAC |
| SC-MH-F | Mycoplasma hominis | GGAAGATATGTAACAAAAGAAGGTGCTG |
| SC-MH-R | Mycoplasma hominis | TTTATCTTCTGGCGTAATGATATCTTCG |

**Supplementary Table 2** Repeatability test result

| Enterprise reference panel | | | CV value (%) | | | CV <5% |
| --- | --- | --- | --- | --- | --- | --- |
|  |  |  | Batch 1 | Batch 2 | Batch 3 |  |
| Nine STD pathogens of enterprise reference panel (plasmid) | CT | CT-R1 | 0.18 | 0.15 | 0.15 | Yes |
|  |  | CT-R2 | 0.15 | 0.16 | 0.19 | Yes |
|  | NG | NG-R1 | 0.12 | 0.12 | 0.12 | Yes |
|  |  | NG-R2 | 0.31 | 0.18 | 0.27 | Yes |
|  | MG | MG-R1 | 0.10 | 0.16 | 0.25 | Yes |
|  |  | MG-R2 | 0.22 | 0.20 | 0.32 | Yes |
|  | TV | TV-R1 | 0.24 | 0.14 | 0.17 | Yes |
|  |  | TV-R2 | 0.22 | 0.25 | 0.37 | Yes |
|  | HSVI | HSVI-R1 | 0.16 | 0.21 | 0.19 | Yes |
|  |  | HSVI-R2 | 0.17 | 0.19 | 0.27 | Yes |
|  | HSVII | HSVII-R1 | 0.09 | 0.16 | 0.31 | Yes |
|  |  | HSVII-R2 | 0.09 | 0.07 | 0.11 | Yes |
|  | UU | UU-R1 | 0.19 | 0.09 | 0.06 | Yes |
|  |  | UU-R2 | 0.19 | 0.00 | 0.11 | Yes |
|  | UP | UP-R1 | 0.10 | 0.16 | 0.15 | Yes |
|  |  | UP-R2 | 0.18 | 0.08 | 0.13 | Yes |
|  | MH | MH-R1 | 0.30 | 0.23 | 0.25 | Yes |
|  |  | MH-R2 | 0.29 | 0.50 | 0.30 | Yes |
| Nine STD pathogens of enterprise reference panel (clinical specimens) | CT | CT-R1 | 0.09 | 0.22 | 0.18 | Yes |
|  |  | CT-R2 | 0.11 | 0.11 | 0.15 | Yes |
|  | NG | NG-R1 | 0.15 | 0.09 | 0.17 | Yes |
|  |  | NG-R2 | 0.19 | 0.17 | 0.19 | Yes |
|  | MG | MG-R1 | 0.10 | 0.13 | 0.18 | Yes |
|  |  | MG-R2 | 0.18 | 0.18 | 0.16 | Yes |
|  | TV | TV-R1 | 0.31 | 0.25 | 0.19 | Yes |
|  |  | TV-R2 | 0.41 | 0.28 | 0.28 | Yes |
|  | HSVI | HSVI-R1 | 0.09 | 0.09 | 0.29 | Yes |
|  |  | HSVI-R2 | 0.00 | 0.15 | 0.15 | Yes |
|  | HSVII | HSVII-R1 | 0.00 | 0.09 | 0.00 | Yes |
|  |  | HSVII-R2 | 0.17 | 0.13 | 0.09 | Yes |
|  | UU | UU-R1 | 0.08 | 0.00 | 0.19 | Yes |
|  |  | UU-R2 | 0.17 | 0.15 | 0.15 | Yes |
|  | UP | UP-R1 | 0.13 | 0.19 | 0.18 | Yes |
|  |  | UP-R2 | 0.08 | 0.13 | 0.08 | Yes |
|  | MH | MH-R1 | 0.32 | 0.22 | 0.22 | Yes |
|  |  | MH-R2 | 0.19 | 0.28 | 0.28 | Yes |

**Supplementary Table 3** Effects of different DNA extraction kits on test results

| STD specimens | Target genes | TM value | | | Consistent |
| --- | --- | --- | --- | --- | --- |
|  |  | Axygen | TIANGEN | CWbio |  |
| Positive STD specimens | CT | 57.8 | 57.8 | 58 | Yes |
|  | NG | 54.3 | 54.7 | 55 | Yes |
|  | MG | 66.7 | 66.5 | 66.7 | Yes |
|  | TV | 66.5 | 67.5 | 67.2 | Yes |
|  | HSVI | 68.8 | 69 | 69 | Yes |
|  | HSVII | 73.5 | 73.8 | 73.8 | Yes |
|  | UU | 55.8 | 56 | 56.2 | Yes |
|  | UP | 66 | 65.8 | 66 | Yes |
|  | MH | 62.8 | 62.5 | 63 | Yes |
| Negative STD specimens | CT | No | No | No | Yes |
|  | NG | No | No | No | Yes |
|  | MG | No | No | No | Yes |
|  | TV | No | No | No | Yes |
|  | HSVI | No | No | No | Yes |
|  | HSVII | No | No | No | Yes |
|  | UU | No | No | No | Yes |
|  | UP | No | No | No | Yes |
|  | MH | No | No | No | Yes |

Note: “No”: No target gene detected.
